# Gastroprotective Effects of Oral Glycosaminoglycans with Sodium Alginate in an Indomethacin-Induced Gastric Injury Model in Rats

**DOI:** 10.3390/vetsci10120667

**Published:** 2023-11-23

**Authors:** Sara Traserra, Héctor Cuerda, Adriana Vallejo, Sergi Segarra, Roger Sabata, Marcel Jimenez

**Affiliations:** 1 Department of Cell Biology Physiology and Immunology, Universitat Autònoma de Barcelona, 08193 Cerdanyola del Vallès, Spain; sara.traserra@uab.cat (S.T.); hector.cuerda@autonoma.cat (H.C.); adriana.vallejo@uab.cat (A.V.); 2 R&D Bioiberica S.A.U., 08950 Esplugues de Llobregat, Spain; ssegarra@bioiberica.com (S.S.); rsabata@bioiberica.com (R.S.); 3 Centro de Investigación Biomédica en Red de Enfermedades Hepáticas y Digestivas (CIBERehd), Instituto de Salud Carlos III, 28029 Madrid, Spain

**Keywords:** glycosaminoglycans, gastroprotection, indomethacin, chondroitin, hyaluronate, dermatan sulfate, sucralfate, sodium alginate, N-acetylglucosamine

## Abstract

**Simple Summary:**

Gastroprotectant drugs tend to be overused in companion animals, as they are employed for various conditions, including gastrointestinal issues caused by medications like pain relievers, which often result in painful stomach ulcers. Our study aimed to discover a safe and efficient method to safeguard the stomach from these detrimental effects. We tested a blend of natural substances known as glycosaminoglycans (GAGs) and sodium alginate in a rat model simulating the gastric damage induced by indomethacin. These substances are recognized for their ability to shield the stomach and promote healing. The outcomes revealed that a combination of sodium alginate, hyaluronic acid, chondroitin sulfate, and N-acetylglucosamine exhibited a significant effectiveness, non-inferior to that of a common gastric medication called sucralfate. This implies that it could serve as an excellent alternative for addressing stomach problems in pets and potentially in humans, reducing the need for other drugs that may have side effects.

**Abstract:**

The gastrointestinal (GI) mucosal barrier is often exposed to inflammatory and erosive insults, resulting in gastric lesions. Glycosaminoglycans (GAGs), such as hyaluronic acid (HA), chondroitin sulfate (CS), and N-acetylglucosamine (NAG) have shown potential beneficial effects as GI protectants. This study aimed to evaluate the gastroprotective effects of oral GAGs in rats with indomethacin-induced GI lesions. Forty-five Sprague–Dawley rats (8–9 weeks-old, 228 ± 7 g) were included in the study, divided into five study groups, and given, administered orally, either sucralfate (positive control group; PC), NAG (G group), sodium alginate plus HA and CS (AHC group), sodium alginate plus HA, CS, and NAG (AHCG group), or no treatment (negative control group; NC). Animals were administered 12.5 mg/kg indomethacin orally 15 min after receiving the assigned treatment. After 4 h, stomach samples were obtained and used to perform a macroscopic evaluation of gastric lesions and to allow histological assessment of the gastric wall (via H/E staining) and mucous (via PAS staining). The AHCG group showed significant gastroprotective improvements compared to the NC group, and a similar efficacy to the PC group. This combination of sodium alginate with GAGs might, therefore, become a safe and effective alternative to prescription drugs for gastric lesions, such as sucralfate, and have potential usefulness in companion animals.

## 1. Introduction

The gastrointestinal (GI) mucosal barrier is continuously exposed to noxious factors leading to the development of inflammatory, erosive and, ultimately, ulcerative disorders [1]. Non-steroidal anti-inflammatory drugs (NSAIDs) are extensively used as anti-inflammatory, analgesic, and antipyretic drugs. However, they are often associated with GI complications in different animal species, especially when used in the long term [2,3,4,5].

NSAIDs inhibit cyclooxygenase (COX), preventing the production of prostaglandins from arachidonic acid. Indomethacin is a non-selective NSAID that blocks both isoenzymes, COX-1 and COX-2 [6,7]. In rats, the administration of indomethacin is a well-established in vivo model for inducing gastric damage [8,9,10].

A wide range of GI protectants are used to treat GI disorders, such as antacids, proton pump inhibitors (PPIs), misoprostol, and sucralfate, and the combined use of mucosal protection with acid suppression has been suggested as an interesting approach to achieving a high rate of GI symptom relief [11,12,13]. While GI protectants can provide significant benefits, it is important to use them judiciously and avoid any potential abuse or overuse. Although generally considered safe, gastroprotectants can have side effects in some cases. Their prolonged or excessive use, particularly in the case of PPI, can increase the risk of certain conditions, such as bacterial overgrowth, micronutrient deficiencies, and bone density issues. One of the most commonly used gastroprotective agents in dogs is sucralfate, a complex salt of sucrose octasulfate and aluminum hydroxide that is widely prescribed for the management of gastric ulcers and other gastrointestinal disorders. Despite being a safe drug for use in dogs, it has the potential to reduce the absorption of other medications, such as ciprofloxacin, theophylline, tetracycline, doxycycline, minocycline, phenytoin, and digoxin [1].

Sodium alginate is a natural polysaccharide that elicits a mucoprotective effect by covering the surface of the gastrointestinal tract and its contents. Accordingly, it has been used as a hemostatic agent to treat GI bleeding from gastric and duodenal ulcers, the erosion of the gastric mucosa, and reflux esophagitis. Its mechanism of action involves the formation of a gel-like barrier in the stomach, which can provide protective and soothing effects. The gel floats on the surface of the stomach contents, forming a physical barrier between the stomach lining and the acidic gastric contents. This barrier helps to prevent the backflow of stomach acid and partially digested food into the esophagus, reducing the occurrence of acid reflux. Moreover, the gel layer created by sodium alginate can act as a protective coating for the stomach lining, shielding it from the corrosive effects of stomach acid [14,15,16].

Glycosaminoglycans (GAGs) are important components of the mucin layer, playing crucial roles in the function and protection of the epithelial surface of the gastrointestinal tract. This layer is primarily composed of mucins, which are large glycoproteins secreted by specialized cells called goblet cells. Mucins consist of a protein backbone with numerous attached carbohydrate chains, predominantly O-linked glycans. These glycans contain various types of sugar residues, including N-acetylglucosamine (NAG), which is one of the repeating disaccharides that, along with glucuronic acid, compose the GAG hyaluronic acid (HA) [17].

Recently, different types of GAGs and NAG have been studied for their potential immunomodulatory and anti-inflammatory effects [18]. GAGs are an heterogenous family of negatively charged polysaccharides. These molecules have important functions in cell attachment, proliferation, migration, and differentiation in many tissues and might be involved in gastric healing. The GAGs chondroitin sulfate (CS), dermatan sulfate, and hyaluronate have been identified in the granulation tissue of skin wounds. These molecules have been shown to inhibit the formation of collagen fibrils and stimulate angiogenesis [19].

The aim of this study was to evaluate the potential gastroprotective effects of different GAG-based formulations in preventing gastric damage in a rat model of indomethacin-induced gastric irritation.

## 2. Materials and Methods

### 2.1. Animals

Fifty-one seven-week-old Sprague–Dawley rats (twenty-five males and twenty-six females) were purchased from Charles River Laboratories France. Rats were between eight weeks old and nine weeks old and had a body weight range of 156.5–315.5 g (227.6 ± 6.297 g) at the time of this study. Animals were maintained in a room with controlled temperature (22 ± 2 °C) and humidity (55 ± 10%). They were housed in cages with a 12 h light–dark cycle (lights on from 07:00 to 19:00 h). Access to food and water was ad libitum, except during the experimental period, in which animals underwent a partial fast of 18 h with access to one pellet and ad libitum water.

### 2.2. Prototype Administration and Induction of Gastric Lesions

Forty-five out of fifty-one animals were randomly divided into 5 experimental groups (n = 9 per group). Each experimental group received different formulations orally, but all of them had a final volume of 0.5 mL. The treatments (Table 1) consisted of: sodium alginate with HA and CS (AHC group); sodium alginate with HA, CS, and NAG (AHCG group), NAG alone (G group), a placebo (negative control group; NC) and a positive control group (PC)—sucralfate (Vetgastril^®^, Opko-Pharmadiet, L’Hospitalet de Llobregat, Spain).

A supplementary group of non-treated animals (n = 6) was established for the comparison of the preservation of the outermost mucus layer of the gastric epithelium. All treatments were formulated so that viscosity was similar in order to avoid a potential bias due to different gastric emptying times that could affect the exposure to indomethacin. Viscosity was measured using a rheometer and expressed in centipoise (cps), using the same conditions of temperature and stirring speed to ensure that conditions were similar between treatments, including the negative control (NC).

Fifteen minutes after the administration of treatment, all animals were administered 12.5 mg/kg of indomethacin orally (Merck, Saint Louis, MO, USA). Four hours after indomethacin administration, animals were euthanased using a guillotine.

The experimental protocol was approved by the Ethics Committee of the Universitat Autònoma de Barcelona (Ref: 2301-CEEA-UAB) and by the Generalitat de Catalunya (Ref: 11270).

### 2.3. Drugs

Indomethacin was dissolved with 1.25% NaHCO_3_ (Panreac Química S.L.U, Castellar del Vallès, Spain) solution to a final concentration of 2.5 mg/mL to induce gastric injury in rats. The temperature was controlled and not allowed to rise further than 37 °C. Solution pH was measured with micropH 2000 (Crison Instruments, Alella, Spain); on average, 2.5 mg/mL of indomethacin had an initial pH of 8.5, which was then adjusted with 100–250 μL of HCl until a final pH of 7.4 was reached, as previously described [20]. The solution was maintained in agitation in a Bibby HC502 magnetic hotplate stirrer until the time of oral administration.

### 2.4. Macroscopic Evaluation

After euthanasia, stomach and duodenal tissues were examined for macroscopic lesions. The stomach of each rat was opened along the greater curvature. It was then carefully stretched and fixed to a silicone plate and photographed with a Nikon camera for further measurements using a computer program (Image J, developed by the National Institutes of Health). A double-blind measurement of the lesions was performed by two independent observers (S.T. and H.C.) to assess and quantify the lesions from the gastric mucosa. The macroscopic assessment consisted of the quantification of the total area with congested mucosa (mm^2^), the percentage of the area with congested mucosa related to the total area of the stomach (%), and the protective effect of the treatments (%). To establish the protective response of each treatment, the following formula was applied [10]:

$$Protective\;effect=\left(\frac{Congested\;mean\;area\;of\;NC\;group-Congested\;mean\;area\;of\;study\;group\;*}{Congested\;mean\;area\;of\;NC\;group}\right)\times 100$$

* AHC, AHCG, G, or PC groups.

A result of 0 is equivalent to no protective effect of the treatment, and 100 equals the total protective effect of the treatment.

### 2.5. Microscopic Evaluation

Stomach and duodenum samples were preserved in 4% formaldehyde for both hematoxylin/eosin (H/E) and periodic acid–Schiff (PAS) staining, which were performed by the Murine and Comparative Pathology Unit (UPMiC) of the Universitat Autònoma de Barcelona. Once the whole sample had been fixed in formaldehyde for 24 h, five sections were obtained per animal and embedded in a histologic cassette, following the protocols described elsewhere [21]. Three sections out of five corresponded specifically to the stomach, and two sections out of five corresponded to 5 cm from the beginning of the duodenum. The fundic sections of the stomach were selected for microscopic evaluation since they showed evident lesions macroscopically. None of the duodenum sections were microscopically evaluated as they did not show gross lesions.

A double-blind examination (by S.T. and H.C.) of the H/E preparations of the stomach was performed via quantifying associated lesions. This measurement was based on two clear and visible alterations: vascular engorgement and submucosal edema. Both microscopic lesions were graded using the scales from 0 to 2 detailed in Table 2 and Table 3, which had been elaborated following advice by the Murine and Comparative Pathology Unit (Universitat Autònoma de Barcelona).

The examination of PAS preparations was conducted via quantifying the mucus thickness of the gastric epithelium. Each slide corresponded to a single specimen, and two gastric fundus sections were collected per animal. The estimations were performed perpendicular to the mucosal surface from the edge of the epithelium to the outermost part of the mucus layer. Three measurements were taken per histological section along its length, one measurement of each edge, and one measurement halfway through the section; therefore, a total of six measurements were obtained per slide. Afterwards, a mean value was obtained. These measurements were based on observation under optic microscopy with a magnification power of 40× associated with an eyepiece graticule corresponding to a size of 1 mm, equivalent to 250 µm for this magnification power.

### 2.6. Statistical Analysis

Data were expressed as mean ± SEM and considered significant when *p* < 0.05. Statistical analysis was performed with GraphPad Prism 6.01 (GraphPad, Software, San Diego, CA, USA). One-way ANOVA was performed for macroscopic and microscopic measurements. Fisher’s post hoc test was used to compare each experimental group with the mean of the NC, or with the non-treated group for mucus thickness data. Before one-way ANOVA, the normal distribution of residuals (D’Agostino and Pearson tests) and the homogeneity of variances (Brown–Forsythe test) was checked.

## 3. Results

### 3.1. Establishment of an In Vivo Model of Indomethacin-Induced Gastric Damage

Increasing concentrations of indomethacin were tested: 6 mg/kg (n = 4), 12.5 mg/kg (n = 4), 18 mg/kg (n = 4), and 24 mg/kg (n = 4). Both macroscopic and microscopic gastric lesions increased with indomethacin in a concentration-dependent manner. The best dose of indomethacin to test gastric protectants was considered to be 12.5 mg/kg. This corresponded to the lowest dose that caused macroscopically and microscopically evident lesions. During the 4-h experimental period, rats were evaluated hourly with regard to behavior, general physical examination, and the Rat Grimace Scale (RGS). None of the study subjects scored higher than 1 at any time for any of the three parameters checked. Therefore, no corrective measures were necessary.

### 3.2. Macroscopic Results

**Figure 1:**
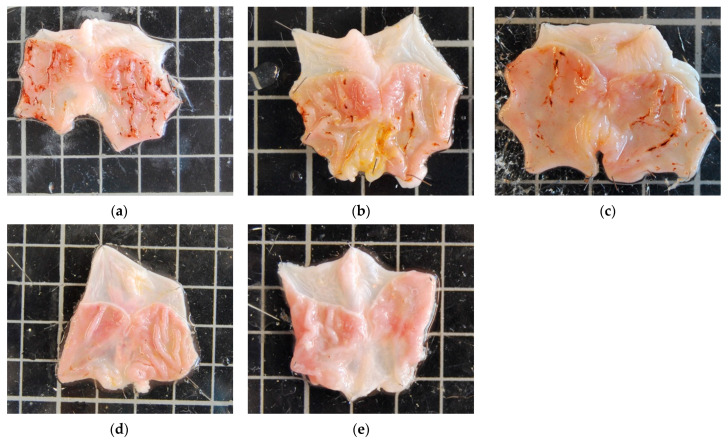
Macroscopic evaluation of the gastric lesions in rats. The NC group (**a**) showed visible congestion of the gastric mucosa. The AHC (**b**) and G (**c**) groups showed moderate injuries. In contrast, almost no gross injuries were observed in the AHCG (**d**) and PC (**e**) groups. For reference, each background grid corresponds to 1 cm^2^ (1 cm length; 1 cm width).

Oral administration of indomethacin at 12.5 mg/kg caused multiple focal lesions in the mucosa of the glandular area of the stomach (Figure 1). The oral administration of treatments in the AHCG and PC groups led to a significant reduction in the extension of the gastric lesion, compared with the NC group, while no significant beneficial effects were seen in the AHC and G groups from that standpoint.

**Figure 2:**
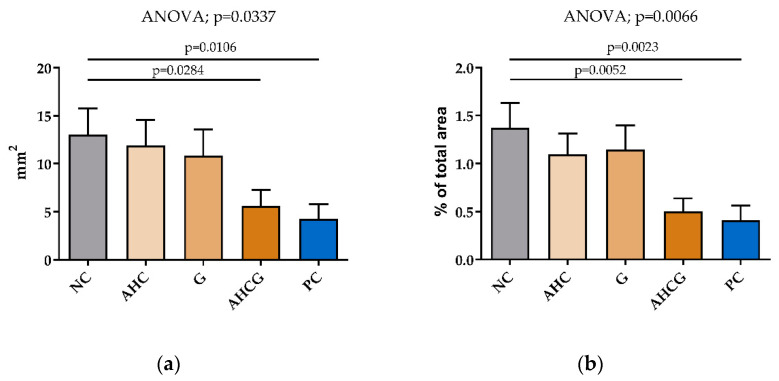
Macroscopic evaluation. Histograms show the total area with congested mucosa (mm^2^) (**a**) and the percentage of area with congested mucosa related to the total gastric area (%) (**b**). All data are presented as mean ± SEM. Fisher’s post hoc test after ANOVA was used to compare each group to the NC group, *p* < 0.05.

When the macroscopic results were related to the total gastric area, a significant reduction in the area with congested mucosa was observed after the administration of treatments in the AHCG and PC groups, while the AHC and G groups did not show a significant reduction in mucosal lesions (Figure 2).

**Figure 3:**
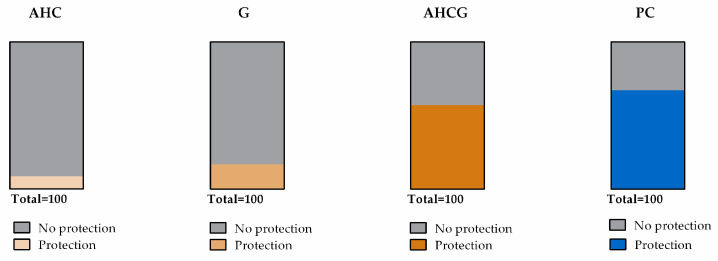
Quantitative representation of gastric protection in the different experimental groups from the total area presenting congested mucosa.

The observed protective response was, from greater to lesser, in the following order: PC (67.20/100) > AHCG (57.03/100) > G (16.85/100) > AHC (8.54/100) (Figure 3). In contrast, no macroscopic lesions were observed in any of the duodenal samples.

### 3.3. Microscopic Results

**Figure 4:**
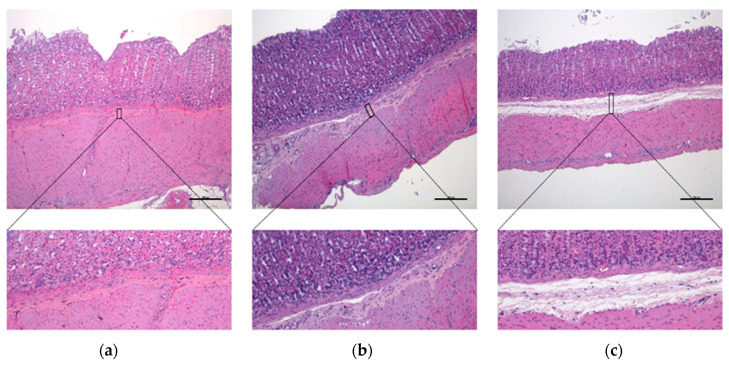
Microscopic comparison of the stablished scores for submucosal edema evaluation. H/E stain, edema score 0 (**a**); H/E stain, edema score 1 (**b**); H/E stain, edema score 2 (**c**). Scale bar, 100 µm.

**Figure 5:**
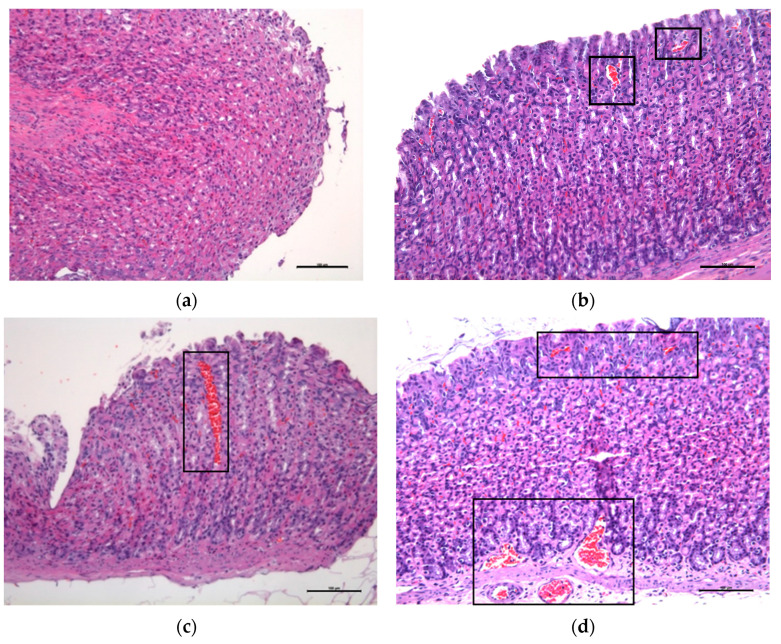
Microscopic comparison of the established scores for vascular engorgement evaluation. H/E stain, vascular engorgement 0 (**a**); H/E stain, vascular engorgement 1 (**b**); H/E stain, vascular engorgement 2 (**c**,**d**). Scale bar, 100 µm.

Histological sections belonging to animals from the NC, AHC, and G groups showed a notable separation of the mucosa and muscular layers and marked submucosal swelling (Figure 4c) plus vascular engorgement penetrating to different depths (Figure 5c,d); while histological sections of groups AHCG and PC showed less severe lesions, consisting of moderate swelling of the submucosal layer, which tended to separate the mucosa and muscular layers, but largely maintained its normal appearance (Figure 4b) plus tortuous vessels (Figure 5b) and mild-to-absent engorgement in submucosal vessels.

**Figure 6:**
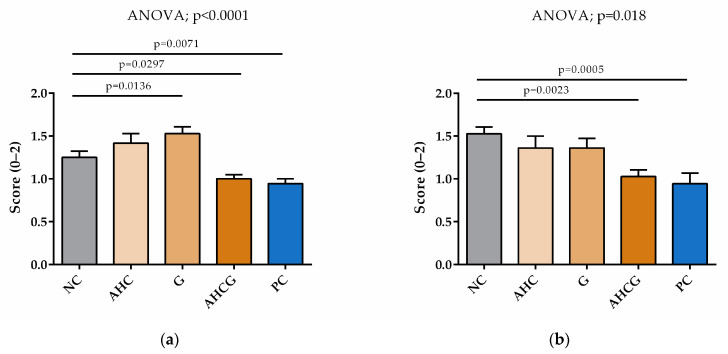
Microscopic evaluation. Histograms showing submucosal edema (**a**) and vascular engorgement (**b**). All data are presented as mean ± SEM. Fisher’s post hoc test after ANOVA was used to compare each group to the NC group, *p* < 0.05.

In the microscopic evaluation, significant differences were observed in the AHCG and PC groups, compared to the NC group, for both submucosal edema and vascular engorgement evaluation (*p* < 0.05). Additionally, significant differences were observed for submucosal edema in the G group, compared to NC. No significant statistical differences were found between AHCG and PC (Figure 6).

**Figure 7:**
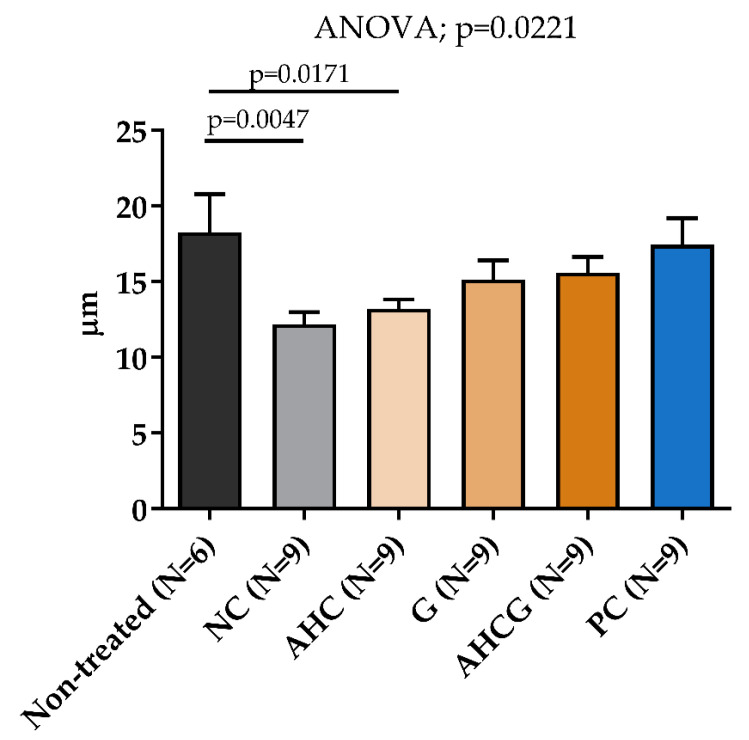
Mucus layer evaluation. Histogram showing the thickness of the mucus layer (µm). All data are presented as mean ± SEM. Fisher’s post hoc test after ANOVA was used to compare each group to the untreated group, *p* < 0.05.

Regarding the preservation of mucus thickness in the gastric areas selected from the macroscopic evaluation, no significant differences were observed in the AHCG, G, and PC groups compared to the non-treated group (Figure 7). Once more, no significant statistical differences were found between the AHCG and PC groups.

## 4. Discussion

Currently, there is a need for novel treatment options to be used in a safe and effective manner as gastroprotectant agents in companion animals in order to reduce the use of some drugs that are often associated with adverse effects. The results from the study herein indicate that a formulation combining GAGs and sodium alginate could be used for this purpose, considering that significant improvements have been achieved in macroscopic and microscopic evaluation through preventing the gastric damage induced with indomethacin, using a methodology similar to that previously employed to investigate the protective effects of sodium alginate in the rat proximal GI tract [16].

In our study, the best gastroprotective effect was achieved in the AHCG group, where animals were administered a novel formulation combining sodium alginate, HA, CS, and NAG. Not only did this group obtain significantly better results than the NC group, it also showed similar results to the PC group, where rats received sucralfate. These data suggest a possible synergistic action between the compounds found in the AHCG combination, as its positive impact was significantly superior to the effects seen in the AHC, G, and NC groups.

Sodium alginate has been reported to elicit a mucoprotective effect by covering the surface of the GI tract and, consequently, inhibiting bacterial translocation. Moreover, it is believed to be effective in both the upper and lower gastrointestinal tracts due to its poor absorption [16]. GAGs and NAG are important components of the mucin layer, playing crucial roles in the function and protection of the epithelial surface of the GI tract. The mucin layer helps to prevent direct contact between the epithelium and potentially harmful substances, such as bacteria, toxins, and digestive enzymes. Additionally, the mucin layer provides lubrication for the movement of food along the intestinal tract [22,23,24]. Furthermore, the mucin contains antimicrobial peptides secreted by enterocytes and Paneth cells, and Hill et al. [25,26] demonstrated that oral HA increased the expression of human β-defensin 2 (hBD-2) and protected against Salmonella infection in vivo and in vitro.

The second layer of protection after the mucin is the intestinal epithelial barrier, whose integrity is guarded by tight junction (TJ) proteins sealing the intercellular space between enterocytes and regulating permeability. These previous findings are key to explaining the added effect of GAGs on the AHCG group. The reinforcement of the mucin layer and an increase in the concentration of TJ, forming a proper barrier of intestinal epithelial cells with less permeability, could be explained by such effects and the beneficial impact of adding GAGs, provided that these improvements might not be possible solely with sodium alginate. It seems that GAGs are able to upregulate the expression and localization of TJ proteins and support the mucin layer, while improving overall barrier function [22,27,28]. Also, the intestinal microbiota plays an important role in regulating biological processes such as epithelial proliferation, mucin production, and antimicrobial compound production [29]. The biological properties of GAGs and NAG have a major impact on the epithelial barrier, reinforcing TJs to avoid a permeability increase, and favoring the proliferation of a beneficial microbiome [24,30,31].

A recent study in neonatal mice observed that oral HA increased the numbers of *Clostridiales* and *Lactobacillales*, which was consistent with findings from Lee et al. [32], who demonstrated that HA bound to bilirubin leads to an increase in the protective bacteria *Clostridium XIVα* and *Lactobacillus* in a DSS-colitis model. A review on CS activity in the gut revealed that there was an association between CS exposure and an increased abundance of genus *Bacteroides* in the murine and human gut [24]. The observed microbiome changes are in line with published data showing an increase in the abundance of *Bacteroides* in dogs with IBD after treatment, which is associated with a healthy microbiome [29]. *Bifidobacterium* are lactic acid bacteria, used as probiotics in humans and dogs, that can reduce the harmful bacterial count and increase short-chain fatty acid (SCFA) levels in healthy companion dogs [33]. There is evidence that NAG can promote the proliferation of *Bifidobacterium* and regulate the gut microbiome [30]. The bacteria enhanced by GAGs contained in the selected formula can increase the levels of SCFA, which are microbial metabolites essential for gut health that nourish the intestinal epithelium and strengthen the mucin layer by increasing the expression of mucins, for example, mucin 2 [34]. Among SCFA, acetate, propionate, and butyrate are the most representative, contributing in a ratio of 3:1:1. These molecules, mainly butyrate, promote the integrity and permeability of the gut barrier in different ways, such as increasing the concentration of TJ, strengthening the mucin layer, and modulating oxidative stress through the restoration of glutathione levels [35].

Sodium alginate, GAGs, and NAG offer a double mechanical protection, creating a gel-like barrier, covering the surface of the gastric content and stomach walls, and strengthening the mucin layer. In addition to mechanical protection, previous studies have shown that the endogenous synthesis of GAGs is increased during wound healing and may be involved in several mechanisms associated with ulcer repair [19]. In the present study, the highest protection was obtained with the compound containing sodium alginate, CS, HA, and NAG, and prior publications report the immunomodulatory and anti-inflammatory effects of CS [18]. Therefore, it has been suggested as a potential treatment for inflammatory bowel diseases [36]. Consistent with our results, CS has previously been shown to exert protective activity in ethanol-induced gastric lesions in rats [37]. Moreover, in a recent case report, a patient with Cameron’s lesions (erosions and ulcers on the ridges of the gastric mucosal folds) with 40 years of evolution did not respond to PPI, but the combination of HA and CS with PPI led to the complete healing of Cameron’s lesions [38]. As previously also described for non-erosive reflux disease (NERD) [11,12,13], these results suggest that the combination of PPI with a mucosal protection therapy consisting of GAGs is more effective than conventional treatments in healing GI lesions. Furthermore, NAG was reported to reduce biofilm formation in invasive *Escherichia coli* strains by interfering with its adhesion to epithelial cells and following colonization [39]. Indeed, the administration of NAG has been shown to improve inflammatory bowel disease in both animal models [27] and humans [40].

Despite the data presented herein, at this point, there are some limitations to be pointed out. First, the current study used an indomethacin-induced GI model, which typically leads to lesions affecting solely the gastric region. Since some of the ingredients included in this formulation have previously been shown to exert beneficial effects against IBD, it would be interesting to focus future studies on the potential impact of such intervention accordingly, by using a colitis model, such as the dextran sodium sulfate (DSS) model. On the other hand, given the importance of the protective mechanism of enhancing the intestinal barrier function, the use of an in vivo permeability tracker such as FITC-dextran could be of help to evaluate GI leakage (oral absorption) between different treatment groups in future studies.

Further investigations are still required to further support the usefulness of this intervention in the target species, i.e., dogs and cats, and even in humans, bearing in mind that this formula has a good safety profile.

The results suggest that this combination of GAGs with sodium alginate is a promising protective agent that could become a safe alternative to prescription drugs for gastric lesions in companion animals. In fact, this product shows promise in terms of use in conjunction with other therapies or even on its own, in certain situations. It should allow a reduction in the dose of, and contribute to minimizing the overuse of gastroprotective drugs such as acid suppressants or sucralfate, which increase the pH and, therefore, may favor biofilm formation that could eventually cause an ulcer.

## 5. Conclusions

The oral administration of a combination of GAGs and sodium alginate in an indomethacin-induced model of gastric injury in rats presents significant gastroprotective effects, which are similar to those of a sucralfate. Although further studies are warranted, this formulation holds great potential to manage GI issues in companion animals, possibly as an alternative to the currently available therapies.

## Figures and Tables

**Table 1:** Composition of the treatments.

Group	Composition
AHC	Sodium alginate, Chondroitin sulfate, Hyaluronic Acid
AHCG	Sodium alginate, Chondroitin sulfate, Hyaluronic Acid, N-acetylglucosamine
G	N-acetylglucosamine, Xanthan gum (E-415), Potassium sorbate (E-202), Citric acid (E-330)
NC	Xanthan gum (E-415), Potassium sorbate (E-202), Citric acid (E-330)
PC	Sucrose sulfate complex with aluminum hydroxide gel (Sucralfate)

**Table 2:** Scale from 0 to 2 to assess submucosal edema. The requirements for each grading are specified.

Scale	Submucosal Edema
0	Normal appearance
0.5	Mild separation of the mucosa and muscular layers.
	The submucosal tissue maintains its normal aspect. Submucosal vessels surrounded by submucosal connective tissue.
1	Moderate swelling of the submucosal layer (edema) separating the mucosa and muscular layers.
	The submucosal tissue maintains its normal appearance. Submucosal vessels surrounded by submucosal connective tissue.
1.5	Important and significant swelling of the submucosal layer (edema). Notable separation of the mucosa and muscular layers.
	Submucosal vessels isolated from other structures.
2	Severe swelling and expansion of the submucosal layer (edema). Maximum separation of the mucosa and muscular layers.
	Submucosal vessels completely isolated from other structures.

**Table 3:** Scale from 0 to 2 to assess vascular engorgement. The requirements for each grading are specified.

Scale	Vascular Engorgement
0	Normal appearance
0.5	Microvessels (capillaries) become slightly tortuous, erythrocytes (RBCs) visible inside.
	Mild increase in the total quantity of erythrocytes.
	Submucosal vessels are not engorged.
1	Tortuous microvessels, RBC visible inside.
	Moderately increased RBC.
	Mild engorgement of the capillaries.
	Some submucosal vessels are mildly engorged.
1.5	Tortuous microvessels (capillaries), RBC visible inside. Important increase in the number of RBC.
	Focal areas of moderately engorged capillaries in the surface layers of the mucosa.
	Some deep engorged microvessels penetrate to the superficial layers.
	Submucosal vessels are engorged.
2	Tortuous microvessels, RBC visible inside, and some extravasation of RBC can be seen.
	Severe increase in the number of RBC.
	Severely engorged capillaries concentrated in the superficial layers of the mucosa. Some deep engorged microvessels penetrate to the more superficial layers.
	Submucosal vessels are engorged.

## Data Availability

The datasets used and/or analyzed during the current study are available from the corresponding author on reasonable request.
